# The Gut Microbial Bile Acid Modulation and Its Relevance to Digestive Health and Diseases

**DOI:** 10.1053/j.gastro.2023.02.022

**Published:** 2023-02-24

**Authors:** Kelly A. Fogelson, Pieter C. Dorrestein, Amir Zarrinpar, Rob Knight

**Affiliations:** 1 Biomedical Sciences Graduate Program, University of California San Diego, La Jolla, California; 2 Skaggs School of Pharmacy and Pharmaceutical Sciences, University of California San Diego, La Jolla, California; 3 Department of Pediatrics, University of California San Diego, San Diego, California; 4 Center for Microbiome Innovation, University of California San Diego, San Diego, California; 5 Division of Gastroenterology, Jennifer Moreno Department of Veterans Affairs Medical Center, San Diego, California; 6 Division of Gastroenterology, University of California San Diego, San Diego, California; 7 Institute of Diabetes and Metabolic Health, University of California San Diego, San Diego, California; 8 Department of Bioengineering, University of California San Diego, San Diego, California; 9 Department of Computer Science and Engineering, University of California San Diego, San Diego, California

**Keywords:** Microbiome, Metabolome, Bile Acid, Engineered Native Bacteria, Short-Chain Fatty Acid, Irritable Bowel Syndrome, Gut–Brain Axis

## Abstract

The human gut microbiome has been linked to numerous digestive disorders, but its metabolic products have been much less well characterized, in part due to the expense of untargeted metabolomics and lack of ability to process the data. In this review, we focused on the rapidly expanding information about the bile acid repertoire produced by the gut microbiome, including the impacts of bile acids on a wide range of host physiological processes and diseases, and discussed the role of short-chain fatty acids and other important gut microbiome–derived metabolites. Of particular note is the action of gut microbiome–derived metabolites throughout the body, which impact processes ranging from obesity to aging to disorders traditionally thought of as diseases of the nervous system, but that are now recognized as being strongly influenced by the gut microbiome and the metabolites it produces. We also highlighted the emerging role for modifying the gut microbiome to improve health or to treat disease, including the “engineered native bacteria” approach that takes bacterial strains from a patient, modifies them to alter metabolism, and reintroduces them. Taken together, study of the metabolites derived from the gut microbiome provided insights into a wide range of physiological and pathophysiological processes, and has substantial potential for new approaches to diagnostics and therapeutics of disease of, or involving, the gastrointestinal tract.

Reductions in data acquisition costs of DNA sequencing^1^ and mass spectrometry (MS), together with improved bioinformatics pipelines,^2–4^ have led to an expanded number of studies performing functional characterization of the gut microbiome. These functional characterization methods, going beyond the taxonomic inventories traditionally produced by microbiome studies, include shotgun metagenomics (characterizing total DNA), metatranscriptomics (RNA), metaproteomics (proteins), and metabolomics (small molecules). Although these new “omics” approaches have expanded our understanding of how the gut microbiome can potentially affect host physiology, they largely remain correlational and hypothesis generating.

Omics studies have shown that the gut microbiome contributes to the pathogenesis of numerous diseases.^5,6^ However, it is unclear whether most therapies that target microbiome composition detectably impact the gut microbiome or are robust to the interpersonal diversity and plasticity of the microbiome in human hosts.^7,8^ Furthermore, many different gut microbiota configurations can lead to the same functional result,^6,9^ suggesting that microbial functions may be more important than composition. To develop a better mechanistic understanding of the microbe–host relationship and more effective microbiome-mediated therapies, a different approach stressing the functional modulation of the gut microbiome is necessary.^10,11^

In contrast to functional characterization methods that elucidate the functional potential of the gut microbiome (ie, shotgun metagenomics and metatranscriptomics), there are 2 omics approaches that provide direct insight into the functional outputs and activity of the gut microbiome: metaproteomics and metabolomics. In this review, we focused on the latter. Research into the gut microbiome’s metabolome allowed us to understand mechanistically how the gut microbiome affects the etiology and pathogenesis of gastrointestinal (GI) disorders. Analyzing the response of the gut microbiome metabolome to defined interventions and using them to build predictive models that apply to individual patients, including re-engineering their microbiomes through the introduction of modified native bacteria,^12^ holds enormous potential. Because the intersection of the microbiome and metabolome is a large topic and recent reviews covered many specific areas (referenced throughout), in this review, we focused primarily on the role of bile acids (BAs) (Figure 1) and on diseases other than inflammatory bowel disease (IBD), which has been covered well in other recent reviews.^13,14^ Our goal was to provide an appreciation of recent works that link the microbiome and metabolome in the gut and relate these metabolites to disease processes throughout the body.

## Key Microbially Mediated Metabolites

**Figure 1.**
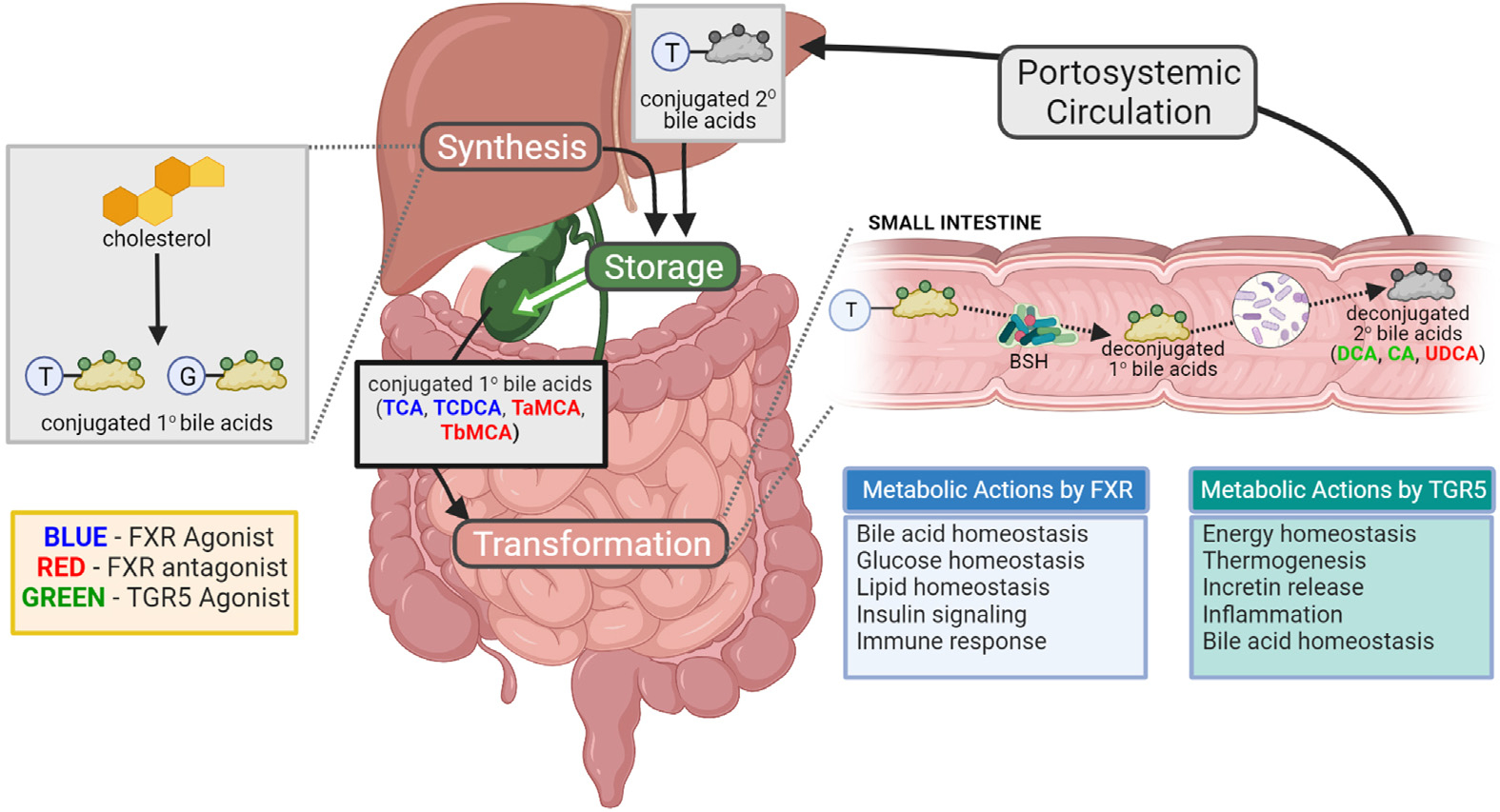
Bacterial BA biotransformations. Cholesterol is converted to primary BAs in the liver. Primary BAs are conjugated with primarily taurine in mice or glycine in humans before being transported to the gallbladder for storage in the form of bile. On ingestion of dietary fats, primary conjugated BAs (within bile) are released into the gut lumen to aid lipid absorption. Bacteria with BSH deconjugate BAs, thereby weakening their soap-like qualities. This allows other microbiome members to further modify them into secondary BAs. Some secondary BAs can be transported back to the liver, where they are then conjugated. The interaction between the gut microbiome and BAs leads to modulation of FXR and TGR5 agonists and antagonists, and thus, allows the gut microbiome to affect host metabolism. T, taurine; G, glycine. In humans: TCA, taurocholic acid; TCDCA, taurochenodeoxycholic acid. In mice: T*α*MCA, tauro-*α*-uricholic acid. Created with BioRender.com.

**Figure 2.**
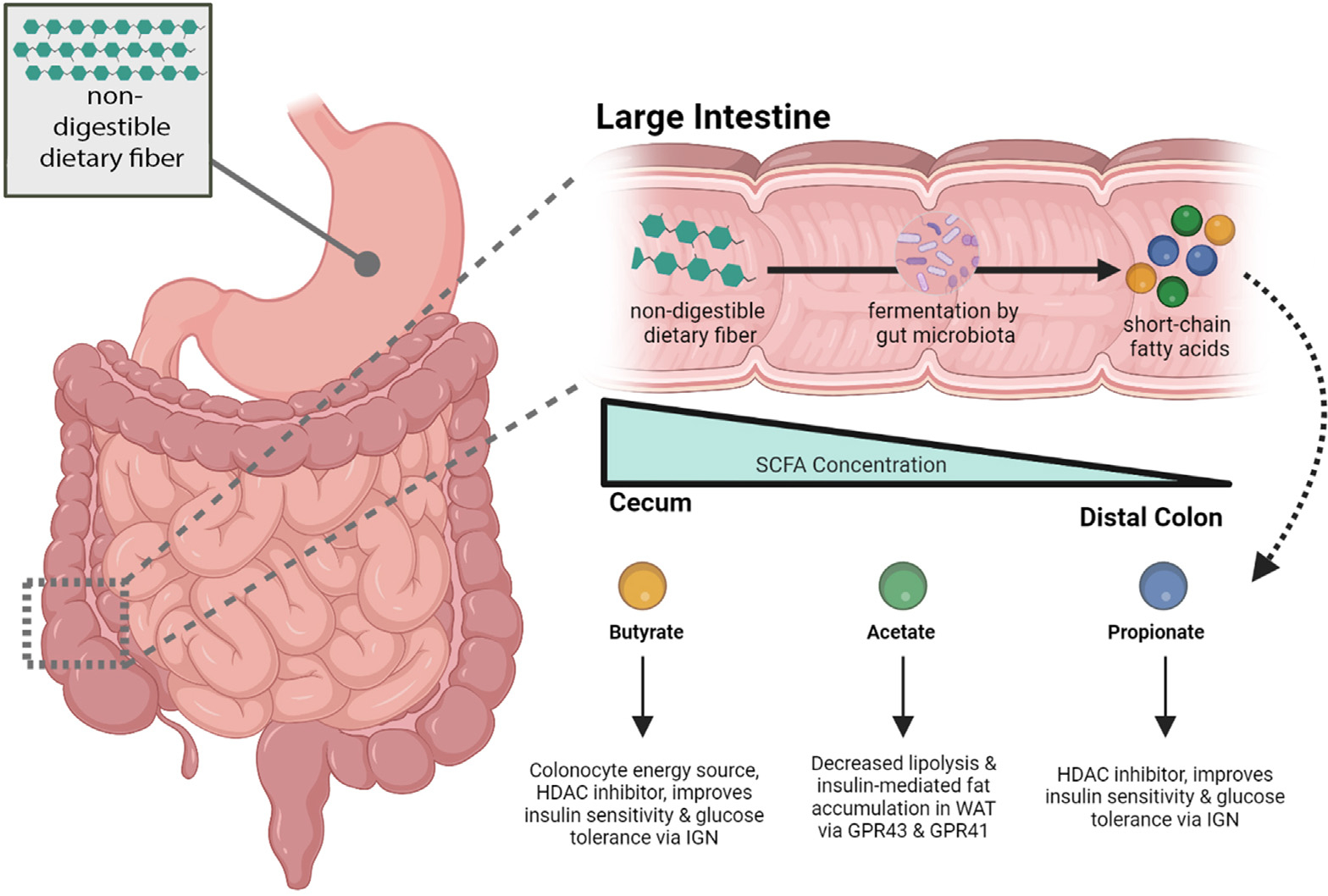
Microbially produced SCFAs and their key effects on host metabolism and digestive disease processes. Butyrate, acetate, and propionate are the 3 main SCFAs produced when gut microbes ferment nondigestible dietary fiber in the large intestine. The cecum and proximal colon have the highest concentration of SCFAs, with a decreasing concentration toward the distal colon. The key roles that the 3 main SCFAs play in processes related to digestive diseases and host metabolism that are highlighted in the article are described. GPR43, G protein–coupled receptor 43/free fatty acid receptor 2; GPR41, G protein–coupled receptor 41/free fatty acid receptor 3); IGN, intestinal gluconeogenesis. Created with BioRender.com.

Two specific classes of bacterial metabolic functions appear repeatedly as important across many studies and physiological systems: BA biotransformations (Figure 1) and short-chain fatty acid (SCFA) production (Figure 2); the former is generating tremendous interest at present due to the unexpected discovery through untargeted MS of many new BAs over the past 2 years.^15–20^ Both BAs and SCFAs have highly disparate diurnal fluctuations, particularly BAs in the ileum,^21^ and are potential entrainment signals of intestinal and hepatic circadian rhythms.^22–24^ Thus, they can have an outsized role in a wide array of homeostatic and physiological processes, as well as conditions in which circadian rhythms have a pathophysiological role, including cancer, aging, inflammation, and metabolism.^25–27^ However, these diurnal variations are poorly accounted for in the published studies and could contribute to often contradictory results in the role of BAs in disease processes, as well as affect the replication of studies. Nevertheless, the importance of BAs and SCFAs as environmental, nutritional, and microbiome-mediated signals cannot be overstated. We therefore focused mainly on new discoveries involving BAs, reviewed SFCAs due to their importance, then provided a brief overview of some of the other important metabolites and references to recent reviews that cover these topics in more detail.

### Bile Acids

Unconjugated and secondary BAs form a key link between luminal bacteria and numerous host metabolic processes. BA signaling pathways, including the nuclear hormone receptor farnesoid X receptor (FXR)^28^ and the G protein-coupled BA receptor 1 (TGR5),^29^ are potent metabolic regulatory pathways that are highly conserved between mouse models and humans (Table 1). BAs also activate other nuclear hormone receptors, including pregnane X receptor, constitutive androstane receptor, vitamin D receptor, liver X receptor *α* and *β* (LXR*α*/*β*, NR1H3), ROR*γ*t, and G-protein-coupled receptors, including S1PR2,^14^ broadening their functional reach. FXR is most highly expressed in the liver, ileum, and kidneys, and to a lesser extent in peripheral tissues, such as the heart, ovary, thymus, eye, spleen, immune cells, neural tissue, and testes.^30^ Although FXR has broad impacts on host metabolic processes, its most well-studied roles relate to its regulation of primary BA synthesis from cholesterol by the liver.^28^ Although FXR regulation of lipid and glucose regulation have been better described in the previous decade,^30–33^ its influence on other physiological systems, such as the blood–brain barrier,^34,35^ and reproduction^36,37^ have only recently been described. TGR5 is found in intestinal L cells, immune cells such as Kupffer cells, and muscle and brown adipose tissue (BAT).^30^ In addition, TGR5 is highly expressed in the gallbladder, lungs, spleen, liver, bone marrow, and placenta.^30^ Broadly, BA-activated TGR5 in peripheral tissues is most well studied for its role in modulating host energy homeostasis.

The BA signaling cycle is initiated in the liver (Figure 1). Conjugated BAs, synthesized from cholesterol and released by the liver, are vital for micelle formation, lipid solubilization and absorption, and cholesterol homeostasis.^38,39^ BAs are produced in the liver by de novo conversion of cholesterol to cholic acid (CA) and chenodeoxycholic acid (CDCA) by CYP7A1. In rodents, CDCA is further metabolized to muricholic acids.^30^ Before excretion into bile, BAs are primarily conjugated with taurine or glycine in humans. Recently, many new conjugated BAs and microbes that conjugate or deconjugate them have been discovered through the combination of untargeted MS-based metabolomics, genome sequencing, and laboratory experiments on individual strains.^15–20^

Because of their detergent properties, BAs can damage bacterial cell walls and modify the microbiome by restricting growth or survival of specific bacterial taxa. Many gut bacterial species in the proximal small intestine are BA-resistant or have developed strategies to modify BAs to protect themselves.^39,40^ Bile salt hydrolase (BSH), an enzyme specific to bacteria, deconjugates BAs and thereby weakens their detergent properties.^41^ Although the host has a dedicated BA transporter for conjugated BAs, the apical sodium–BA transporter, deconjugated BAs do not have transporters and are reabsorbed with more difficulty through passive diffusion.^30^ Thus, bacterial deconjugation of primary conjugated BAs promotes the excretion or retention of deconjugated BAs in the lumen to the distal colon, where other bacteria can use them as substrates. Deconjugated BAs become available for further biotransformations by other bacteria, yielding secondary BAs, including deoxycholic acid (DCA), ursodeoxycholic acid (UDCA), and lithocholic acid.^40,42^ Although much of this hydrophobic pool of secondary BAs is excreted, enough is absorbed through passive diffusion to change the serum BA pool and act as signaling molecules.^43^ These BAs act as agonists (eg, DCA and lithocholic acid for TGR5 and CDCA for FXR) and antagonists (eg, tauro-*β*-muricholic acid [T*β*MCA] for FXR) to BA receptors (Figure 1). Deconjugated and secondary BAs are absent in germ-free mice,^44–47^ and heavily decreased in antibiotic-treated, microbiome-depleted mice,^48^ proving that the microbiome produces them.

BAs have a profound impact on many aspects of mammalian physiology and disease, not limited to the GI tract. In metagenomic studies of human populations and mouse models, BSH is potentially protective against obesity, dysmetabolism, and many other physiological disturbances.^49–51^ Conjugated, deconjugated, and secondary BAs serve as important signaling molecules to affect many physiological processes,^30,38^ including cholesterol,^52,53^ lipid,^54^ and glucose homeostasis^54–61^; blood–brain barrier permeability^34^; neuroinflammation^62,63^; circadian rhythms^22,24,64^; and neurodegeneration.^65^ The distribution and variety of BA receptors (ie, FXR, TGR5, vitamin D receptor, PAR, constitutive androstane receptor, ROR*γ*t, and SP1R2) among mammalian cells suggests that BAs could have an even greater role in host physiology than initially imagined.^30^ BA signaling pathways are viable therapeutic targets that should be investigated for a variety of diseases, including IBD, pancreatic insufficiency in cystic fibrosis, and nonalcoholic fatty liver disease (NAFLD) in humans_._^15,18,19,66^

Glucose homeostasis is a key physiological process affected by BAs. BAs can affect insulin resistance through FXR and TGR5.^43^ FXR modulates, either directly or through fibroblast growth factor (FGF-15 or FGF-19 in humans), scores of genes that are involved in metabolic homeostasis.^67^ For example, murine studies demonstrate that FXR modulates the release of glucagon-like peptide 1, an insulinotropic GI hormone/incretin that regulates insulin secretion^68^; gluconeogenesis in liver and muscle by reducing expression of phenol pyruvate carboxy kinase, glucose-6-phosphatase, and fructose-1,6-biphosphatase^69^; and browning of white adipose tissue (WAT).^70^ In addition, murine studies demonstrate that, compared with conventionally raised wild-type (WT) mice fed a high-fat diet (HFD), FXR knockout mice fed HFD do not experience diet-induced weight gain or hepatic steatosis, demonstrating the requirement of FXR signaling in mediating diet-induced dysmetabolism and glucose dysregulation.^60^ TGR5 is found in intestinal L cells, immune cells such as Kupffer cells, and muscle and BAT. Fat stored in BAT is used to generate heat. BAT is most abundant in humans during infancy and decreases with age. In L cells, TGR5 activation primarily affects glucose homeostasis through the secretion of glucagon-like peptide 1. In addition, TGR5 affects overall metabolic homeostasis by increasing energy expenditure in BAT and muscle by converting thyroxine to triiodothyronine without changing circulating thyroid hormone levels.^43^ Nevertheless, the relationship of the gut microbiome and TGR5 signaling is not completely understood.

BA signaling is affected by diet. Although FXR has been investigated in different animal models of dysmetabolism, studies have generated conflicting results and the role of BAs in metabolism remains controversial.^30^ FXR-deficient mice on a normal chow diet develop hyperglycemia and hypercholesterolemia.^61,71^ However, as mentioned above, FXR-deficient mice on HFD are protected against obesity and exhibit improved glucose homeostasis.^56,60^ Diet and the time of sample collection possibly contribute to the different phenotypes observed in these experiments.^30^ A more extensive review on the effects of diet on BA signaling in mice can be found in Li and Chiang.^72^

Sex is an important biological variable that affects BA signaling. Mouse and human studies have repeatedly demonstrated sexual dimorphism in fecal, serum, and gallbladder BA composition.^73–77^ In mice, females have a significantly larger total BA pool, as well as higher serum concentrations of total, primary, and secondary BAs.^73,74^ Given the structural similarity between BAs and sex steroids, physiologically significant cross-talk may occur between these 2 systems. Some androgens, such as androsterone, can activate FXR receptors and influence BA synthesis and composition.^78^ Conversely, BAs can influence plasma testosterone concentrations to even affect host fertility.^79,80^ It is unclear whether BAs affect female reproductive hormones and fertility. Nevertheless, these studies suggest that the gut microbiome can influence fertility and reproduction either directly through FXR and TGR5 or through BA cross-reactivity with sex hormones.

There are active research areas investigating synthetic ligands for BA receptors such as FXR and TGR5. However, studies have arrived at differential results. For example, 2 murine studies published in the same year found opposite effects when investigating the effects of intestine-specific FXR agonism vs antagonism by synthetic ligands on obesity-related metabolic dysfunction; in 1 study, intestine-specific FXR agonism with fexaramine improved obesity-related metabolic dysfunction, such as weight gain and insulin resistance,^70^ whereas in the other study, intestine-specific FXR antagonism with glycine-*β*-muricholic acid reportedly had the same therapeutic effects.^81^ Whether these differences are the result of different techniques (eg, mode of administration) or off-target effects is unclear and warrants additional investigation. A thorough review on natural and synthetic ligands that have been developed to target FXR can be found in Jiang et al^82^ and Carotti et al.^83^

### Short-Chain Fatty Acids

SCFAs are produced when gut microbes ferment dietary fibers into butyrate, acetate, and propionate in the large intestine (Figure 2).^84^ SCFAs can also be derived from microbial fermentation of protein, although this process primarily gives rise to branched chain amino acids.^85^ Conversion of dietary fiber into SCFAs involves enzymatic reactions distributed broadly among gut bacterial taxa (see Koh et al^85^ for a thorough review). The pathways and precursors that gut microbiota use to derive SCFAs are adaptable to nutritional changes, enabling maintenance of essential SCFA levels despite nutritional variation. For example, butyrate can be synthesized by protein via the lysine pathway, although it is most commonly formed by acetyl coenzyme A precursors via the acetyl coenzyme A pathway.^86^ Furthermore, an in vitro study demonstrated that fecal-derived microbiota communities from human donors adaptably produce SCFAs in response to incubation with different nondigestible carbohydrates. Specific bacteria were stimulated in response to particular carbohydrate substrates and, overall, SCFA production was reproducible in response to these substrates, despite interindividual differences in gut microbiota composition.^87^

SCFAs have a broad impact on numerous host processes, ranging from physiology to gene expression. There are 2 main signaling mechanisms through which SCFAs affect host physiology: histone deacetylase (HDAC) inhibition and G-protein coupled receptor signaling. SCFAs, particularly butyrate and propionate, function as HDAC inhibitors and have the potential to broadly impact gene expression in the host. HDAC inhibitors can affect host immune responses and have anti-inflammatory and immune-suppressive effects and are therefore believed to improve intestinal health. Thus, harnessing the HDAC inhibitory effects of SCFAs for cancer therapeutics is an area of active investigation.^88–90^ Butyrate is the main energy source for colonocytes and is present in high concentrations in the distal lumen, but also protects against inflammation and colorectal cancer (CRC), in part by acting as an HDAC inhibitor.^91^ However, butyrate at high concentrations can, counterintuitively, promote cancer rather than suppress it; cancerous colonocytes use glucose as their primary energy source and accumulate butyrate in order to inhibit HDACs that would otherwise impose normal regulation of gene expression. Accordingly, butyrate concentration was 3-fold higher in nuclear extracts of cancerous cells compared with noncancerous cells.^92^ Furthermore, dietary fat can influence the efficacy of butyrate in preventing tumorigenesis. For example, rats fed butyrate in combination with fish oil had increased apoptosis and decreased cell proliferation in colonocytes compared with rats fed butyrate in combination with corn oil.^93^ Thus, butyrate functions in a cell-type–specific and environment-specific fashion that is highly dependent on concentration, time of exposure (ie, time in tumorigenic process), and interaction with dietary fat.

SCFAs also serve as ligands for numerous G-protein coupled receptors, thereby affecting a wide range of host metabolic processes. Acetate is present in high concentrations in peripheral circulation. Thus, it is capable of reaching peripheral tissues and generally results in beneficial metabolic effects in WAT, brain, and liver. WAT is the predominant form of fat in the body and primarily serves the role of energy storage. In WAT, increased acetate levels are associated with decreased lipolysis and decreased insulin-mediated fat accumulation. Two of acetate’s key receptors are GPR43 and GPR41, which can also detect other SCFAs (Figure 2). GPR43 has been better characterized and in vitro studies have demonstrated that its activity is associated with leptin secretion, adipogenesis, and antilipolytic activity in WAT.^94–96^ However, these experiments did not correctly reflect physiological conditions in vivo. GPR43 knockout mice on a normal chow diet become obese compared with WT mice on the same diet, and mice with adipose-specific overexpression of GPR43 are lean, even on an HFD.^97^ However, obesity-protective effects in the adipose-specific GPR43 overexpression murine model are reversed when mice are given antibiotics, suggesting a role for the gut microbiome in mediating this effect. Stimulation of GPR43 in WAT, but not liver or muscle, suppresses insulin signaling and improves glucose and lipid metabolism.^97^ The effects of GPR41 in WAT are less well characterized, but GPR41 knockout mice are leaner than WT mice. However, this effect is absent in germ-free GPR41 mice,^98^ again suggesting a role for the microbiome in mediating its effect. Propionate and butyrate promote beneficial metabolic effects via intestinal gluconeogenesis, which signals through a gut–portal–brain neural circuit to increase satiety and improve glucose tolerance and insulin sensitivity.^99^ A more extensive review of SCFA–G-protein coupled receptor signaling can be found in Koh et al.^85^

SCFA levels can be influenced by diet. The food that an individual consumes affects the composition of their gut microbiome, and thus has an influence on their unique SCFA profile.^100^ Dietary fiber (in the form of arabinoxylan-oligosaccharides) increases SCFAs in general, and butyrate, in particular, restores beneficial microbes and lowers toxic microbial metabolites.^101^ A study completed on human samples collected postmortem demonstrates that the cecum and proximal colon have the highest concentration of SCFAs, with a decreasing gradient toward the distal colon.^84^ SCFAs are absorbed and drain into the portal vein. Of the three main SCFAs, acetate is the most abundant in peripheral circulation.^84^ Butyrate and propionate are present at lower concentrations in peripheral circulation, as butyrate is used by colonocytes and propionate is metabolized in the liver.

## Affected Diseases

### Nonalcoholic Fatty Liver Disease

NAFLD presents as a spectrum of liver diseases that can generally be grouped into the following categories: nonprogressive simple steatosis and nonalcoholic steatohepatitis (NASH), a progressive form of NAFLD that is characterized by inflammation and hepatocyte injury.^102^ Although nonprogressive simple steatosis carries little risk of advancing to progressive stages, NASH greatly increases the risk of irreversible liver damage and rise in hepatology-related mortality risk from cirrhosis and hepatocellular carcinoma.^103^ Human studies associate NAFLD with compositional changes in the gut microbiome, which have recently been reviewed extensively.^104,105^ Furthermore, a recent study in participants from a prospective twin and family cohort, including 98 probands along the entire NAFLD spectrum and 105 first-degree healthy relatives, demonstrated the efficacy of using gut microbiome–derived signatures to detect NAFLD cirrhosis.^106^ Previous research demonstrated that BA homeostasis is dysregulated during NAFLD.^107,108^ For example, compared with healthy controls, patients with NASH have higher levels of total fecal BAs, CA, CDCA, and BA synthesis, and an increased ratio of primary to secondary fecal BAs.^107^ In a study of individuals with biopsy-proven NAFLD, total unconjugated serum BAs were lower in individuals with NASH and fibrosis, and total serum BAs are elevated during fibrosis when compared with individuals with NAFLD.^108^ Dysregulation of BA homeostasis along the NAFLD spectrum can affect disease pathophysiology via dysregulation of host metabolic processes that are modulated by the BA receptors FXR and TGR5. Importantly, FXR modulates BA,^28^ glucose and lipid homeostasis,^30–33^ as well as immune responses and insulin signaling.^30^ TGR5 plays an important role in energy homeostasis, insulin signaling, and inflammation.^30^ As these metabolic processes are perturbed along the NAFLD spectrum, disruptions in BA homeostasis may perturb these metabolic processes via deregulation of their receptors. The synthetic BA derivative obeticholic acid, an FXR agonist, is being investigated in phase III clinical trials for the treatment of NAFLD and NASH fibrosis.^109^ FXR is a key regulator of BA homeostasis that also regulates inflammation and lipid homeostasis.^30^ The expression of *Fxr* is down-regulated during NASH.^110^ Overall, these findings suggest that NASH may promote a luminal environment with a greater proportion of BAs that function as FXR antagonists, but are likely to also interact with other receptors. Thus, bacterial biotransformations that could remove these antagonists, such as BSH, could play a therapeutic role in treating NASH.

### Functional Gastrointestinal Disorders and Irritable Bowel Syndrome

Despite recent advances in understanding the role of the gut microbiome in functional GI disorders, there have been few advances in the ailments that are responsible for the most common causes of GI ambulatory visits, including abdominal pain, chronic diarrhea, and chronic constipation. These functional GI disorders are likely heterogeneous, grouped together based on shared symptoms rather than endoscopic, radiologic, or blood biomarker diagnostic tests.^111,112^ The underlying pathophysiology of these disorders has been elusive, mainly because of the heterogeneity of the disease, few animal models, and poor physiological means to subclassify patients.^113^ Functional GI disorders involve changes in gut motility, visceral hypersensitivity, intestinal permeability, and intestinal secretions, all of which can affect, and be affected by, the gut microbiome.^114^ Recent gut microbiome studies suggest a role for intestinal microbial environments and alterations of luminal metabolite profiles.^115–118^ The potential mechanisms by which luminal products, some of them bacterial, could affect these physiological pathways are being vigorously investigated.^119,120^ A recent review of the role of microbiome in animal models of visceral pain provides an excellent summary.^121^

Animal models show a clear relationship between the gut microbiome and host phenotype and potentially implicate BAs. Experiments demonstrate that the gut microbiome transplanted from patients with irritable bowel syndrome (IBS), a subset of patients with functional GI disorders, into gnotobiotic mice can modulate intestinal permeability in a manner that is dependent on proteolytic activity of the transplant.^122^ Gnotobiotic mice humanized with high proteolytic activity IBS microbiota from patients with post-infection or constipation-predominant IBS (IBS-C) had greater permeability than those colonized with low proteolytic activity IBS microbiota.^122^ In addition, gut microbiome transplanted from patients with IBS-C can modulate pain-sensation thresholds,^123^ and gut microbiome transplants from patients with IBS-C and diarrhea-predominant IBS (IBS-D) can modulate gut transit time.^124,125^ IBS-D is associated with increased colonic BA exposure, and a rodent study found that BA-induced exacerbation of visceral hypersensitivity is mediated by FXR.^126^

Human studies in IBS have been limited by their cross-sectional design and, at times, lack of subtype classification or assessment of symptoms at the time of sample collection.^127,128^ Although there is no consensus among these studies, they point to several metabolomic changes in patients with IBS, particularly BAs. Changes in fecal BAs were profiled in a study designed to investigate how BA levels relate to symptoms, gut microbiome changes, and diet in women with IBS. Compared with healthy controls, 40% of women with IBS had significantly increased secondary conjugated fecal BAs, including glycodeoxycholic acid, taurodeoxycholic acid, and glycolithocholic acid.^129^ Further subset analysis separating patients into IBS-C, IBS-D, or IBS-mixed demonstrated high secondary conjugated fecal BAs in women with IBS-D and IBS-mixed. Other investigators suggest that a small subset of patients with IBS-D have BA malabsorption (determined by retention of radiolabeled selenium-75 homocholic acid taurine) and that fecal metabolomics could identify this subset.^130^ Primary BAs act as detergents and have antimicrobial properties that can potentially damage bacterial cells by breaking down their membrane bilayer, thus restricting the survival or growth of specific bacterial taxa.^131^ This could explain the reduced diversity observed in the microbiome of patients with IBS-D.^132^ Two studies of patients with IBS-D confirmed an increase in total fecal BAs and decrease in FGF-19 in a subset of approximately 25% of patients.^133,134^ Fecal levels of primary BA (not total fecal BAs) may better identify individuals with BA malabsorption.^134^ This change in BAs was correlated to an increase in *Clostridium* spp. The addition of IBS-D fecal microbiota or *Clostridium scindens* increased fecal BA excretion and decreased FGF-15 in mice, whereas treatment with vancomycin led to the discovery of opposite results.^133^ Thus, the presence of *Clostridium* spp may identify a subset of patients with IBS-D that respond to specific microbiome-mediated treatments.

The most comprehensive study on the role of the gut microbiome and its metabolites on functional GI disorders is a longitudinal study on subsets of patients with IBS and healthy controls.^117^ This study used a multi-omic analysis of stool samples (ie, metagenomics, 3-method metabolomics), colon biopsies (ie, 16S ribosomal RNA, gas chromatography–MS metabolomics, host transcriptomics, methylome), and serum samples (ie, liquid chromatography–MS metabolomics) combined with dietary and disease assessments, biopsy and serum cytokine analysis, and host physiological measurements from the biopsy samples. Because certain BAs can affect intestinal fluid secretion in humans,^135^ the authors analyzed the fecal BA pool in their patient subsets. Patients with IBS-D had higher amounts and patients with IBS-C had lower amounts of unconjugated primary BAs, especially CA and CDCA, in the fecal BA pool, compared with healthy controls. Another case-control study found similar changes in patients with IBS-D.^136,137^ BA changes in the stool increased intestinal secretion, as assessed by ionic fluxes across the epithelium in an Ussing chamber, supporting a role for elevated BA levels in increasing fluid content in patients with IBS-D. Although BA malabsorption may play an important role in driving intestinal secretion in patients with IBS-D, this study suggests that lack of biotransformation of unconjugated primary BAs to secondary BAs may be an important contributing factor to the pathophysiology of this disease. The integration of the microbiome and metabolomic data with transcriptomic and epigenetic characterization of the same patients revealed potentially novel host–microbiome interactions that may be contributing to IBS. These correlational results require further investigation with more mechanistic studies in bedside-to-bench research programs.

BAs were not the only metabolites that were linked to disease pathogenesis. Physiological studies of colon samples from patients with IBS-C demonstrated a decrease in epithelial ion transport and water secretion, with a concomitant decrease in the SCFAs propionate, butyrate, and acetate.^117^ Other investigators have demonstrated this change in SCFAs in cross-sectional studies.^132^ SCFAs can modulate gut motility by affecting the GI serotonergic pathway, primarily by promoting transcription of Tph1 in enterochromaffin cells.^138,139^ Conversely, in patients with IBS-D, investigators observed an increase in tryptophan and tryptamine, 2 bacterial metabolites that can activate gut serotonin receptors and increase fluid secretion^140^ are also increased in patients with IBS-D compared with healthy controls.^117^

### Colorectal Cancer

BAs are intimately involved in CRC, the fourth most commonly diagnosed cancer and the fourth most common cause of cancer deaths in the United States.^141^ Diets rich in foods that have high quantities of animal protein and fat combined with low quantities of fiber are strongly associated with CRC risk.^142,143^ These diets resulted in elevated levels of fecal secondary BAs, particularly DCA.^144,145^ The role of BAs as tumor promoters has been tested using a variety of experimental settings.^146^ More than 85% of CRCs arise from a mutation in the adenomatous polyposis coli gene.^147^ Recent studies found a relationship between the BA receptor FXR and APC.^146^ Increased FXR activity is inversely correlated with CRC progression^146,148,149^; loss of FXR in the APC^Min/+^ mouse model of CRC leads to the development of intestinal tumors.^148^ Suppression of FXR with its antagonist T*β*MCA^150^ leads to CRC progression; reduction of T*β*MCA leads to FXR activation and CRC suppression.^151^ In mice, T*β*MCA can be reduced by increasing luminal BSH activity, a bacterial enzyme that deconjugates BAs,^49^ suggesting possible microbial function that can be involved in tumor progression, as well as playing a therapeutic role in CRC suppression in humans.

### Gut–Brain Axis

Dysbiosis has been associated with changes in social, communicative, stress-related, and cognitive behaviors in murine models.^152,153^ Human studies have linked perturbations in the gut microbiome and autism spectrum disorders,^154^ major depression,^155^ and Parkinson’s disease (PD).^153^ There is growing evidence that microbiome–neuroimmune interactions can mediate behavioral and physiological abnormalities observed in murine models, specifically through global changes in brain transcriptome, altered microglial maturation and function, and integrity of the blood–brain barrier.^156,157^ However, it is not clear what agents, and through what mechanisms, these effects are mediated.

BAs play a particular role in neuroinflammation. Both FXR*α* and TGR5 receptors are found in brain tissue, including microglia and neurons. UDCA, a secondary BA created by bacteria, and its hepatic taurine-conjugated (tauroursodeoxycholic acid [TUDCA]), are immunomodulatory agents that affect microglia. UDCA inhibits the production of the pro-inflammatory cytokine interleukin-1*β* and nitric oxide, and can counteract a neurotoxin’s effects on neuronal death and synaptic changes in vitro.^158,159^ In mouse models of neuropathologies, TUDCA reduced microglial activation, decreased inflammatory cytokines, and preserved neuronal integrity,^65,160^ Although most studies on BAs and neuroinflammation have used UDCA or its glycine or taurine conjugates, it is not clear whether other BAs, especially the recently discovered BAs, have similar effects. The UDCA immunomodulatory effects are mediated through the TGR5 receptor.^63^ In fact, a TGR5 agonist also reduced microglia activation and proliferation and reduced proinflammatory cytokines.^161^ However, other receptors by which BAs can affect neuroinflammation have also been proposed.^62^ Moreover, because BAs and TGR5 activation play an important role in GI immune system and epithelial barrier function,^162–165^ their disruption of the gut barrier, with the ensuing inflammatory reaction, has significant consequences on brain health.^163^

Recent studies in autism spectrum disorders and PD demonstrate this relationship between epithelial integrity, BAs, and brain pathology. In a mouse model of autism spectrum disorders, the pathological behavioral phenotype was associated with impaired epithelial barrier function and deficient BA deconjugation.^166^ However, it is not clear whether the increasing luminal BSH activity could improve the autism spectrum disorders phenotype. This is currently being investigated with probiotics with BSH activity in mouse models and humans. For PD, alteration of secondary BAs is a key finding in patients with Parkinsonism compared with controls.^167–169^ In addition, TGR5 agonism alleviates the inflammatory neurodegeneration in a mouse model of PD^170^ and TUDCA, a TGR5 agonist, improves motor symptoms in a mouse model of PD.^171^ Better methods to more mechanistically study the relationship between bacterial BA modifications and neuroinflammation and neuropathological diseases are necessary to move this field forward.

### Aging

Age-related disorders, including inflammation, neurodegeneration, frailty, and intestinal disorders, are accompanied by significant shifts in the composition of the gut microbiome.^172–177^ The aging microbiome is characterized by a decrease in saccharolytic potential, a decrease in genes vital to SCFA production, and an increase in proteolytic functions.^178^ However, neither the underlying mechanisms causing these compositional shifts nor the metabolic consequences to host health are well understood. Either fecal transplantation or cohousing with younger mice restores normal gut immune function and improves healthspan.^179^ Thus, the gut microbiome is a promising therapeutic target for age-related dysfunction and prompts the hypothesis that changes in host health are driven by systemic changes in the microbial composition or function rather than a single bacterial family.

Aging-related dysmetabolism could be mediated by altered luminal BA signaling, itself mediated by functional changes in the gut microbiome. In humans, aging affects the serum BA pool,^76,180^ with higher levels of taurocholic acid associated with a shorter lifespan independent of any association with cardiovascular disease or cancer.^181^ The mechanisms driving these phenotypic changes and how they contribute to longevity are unknown, but these changes may be due to reduced reabsorption, increase in BA synthesis, modulation from the gut microbiome, or a combination of these. Gut microbes can further metabolize taurocholic acid into hydrogen sulfide and DCA, which are genotoxins and tumor promoters, respectively, and could intensify aging symptoms in the host.^182^ Although 1 study found FXR down-regulation contributes to age-related dysmetabolism, global *Fxr* knockout mice showed decreased adiposity with age and improved insulin sensitivity.^183,184^ Although these studies demonstrated that FXR signaling plays an important role in age-related dysmetabolism, it is not yet clear whether bacterial modulation of BAs affects host metabolic health through these signaling mechanisms, or if modification of the microbiome could modulate aging. However, random forest analyses do show that the microbiome overall is strongly associated with aging, and that a readout of the microbiome can even predict the age of an individual.^176,185^ In addition, healthy aging is correlated with continual drift toward compositional uniqueness and an increase in microbially derived circulating amino acid derivatives, such as phenylacetylglutamine and *p*-Cresol sulfate. Moreover, aging results in a microbial pattern that favors the depletion of core species, such as *Bacteroides*.^186^ It is important to determine whether deviations from this overall trend are correlated with outcomes, such as whether people with microbiomes and/or metabolomes that resemble those of younger individuals for their chronological age are healthier, and whether these outcomes can be manipulated.

## Future Outlook and Conclusions

Despite the investment of effort and money, microbiome-mediated therapies, such as fecal microbiome transplantation, probiotics, prebiotics, fecal capsules, and engineered live bacterial therapeutics, are still limited in their use and efficacy for human digestive diseases.^187–190^ Better functional understanding of not only microbiome compositional changes associated with a disease, but also the functional implications of these compositional changes, can potentially lead to interventions that bypass the microbiome itself and act on receptors that these secondary agents modulate. Current human studies have not been designed to elucidate whether the gut microbiome could account for sex and race differences in the prevalence of various diseases. This will require additional, well-designed studies once potential mechanisms from animal studies or larger clinical trials are identified. Although major advances have been made in the utilization of multi-omic approaches to functionally characterize the gut microbiome, substantial improvements are still needed in the field to improve data interpretation and application in the context of human health and disease. To understand the precise mechanisms by which the gut microbiome contributes to digestive diseases, significant challenges in tracing the specific bacterial origin of microbially produced metabolites will need to be overcome. Advanced analytical techniques are being developed that integrate multiple omic data sets to trace the source of microbially produced metabolites and identify key metabolites associated with disease states.

The discovery of more than 100 new conjugated BAs and microbes that conjugate or deconjugate them demonstrates the complex and ever-evolving nature of characterizing the impact of the gut microbial metabolome on human health and disease.^15,191^ Newly identified BAs include phenyl-alanocholic acid, tyrosocholic acid, and leucocholic acid, which were discovered by analysis of untargeted metabolomics of 29 organs of mice colonized by a normal microbial community in comparison with data from germ-free mice.^15,191^ These BAs were discovered through the application of molecular networking, which enabled the discovery of MS fragmentation spectra to annotated spectra. Molecular networking is a strategy in which similarities between MS/MS spectra are computed, then visualized as a network to find different but related molecules. In this case, the MS/MS spectra of the new conjugates were similar to glycocholic acid, but the glycine had been substituted with the different amino acids. Synthesis confirmed that the BAs were indeed conjugated to cholate and not the expected muricholate, the dominant BA in mice. These BAs were found at a higher frequency in samples from human subjects with IBD or cystic fibrosis than from control samples from the American Gut project.^15^ NAFLD is highlighted in Wang et al,^19^ when the phenylalanine conjugate of DCA matched the most significantly differing spectra between human subjects with NAFLD and controls. Furthermore, a recently developed reverse metabolomics strategy identified 62 novel BA metabolites, which were confirmed to be produced by gut bacteria, to be associated with IBD.^18^ Overall, the implications of these new discoveries are only in the early stages and will take decades to fully appreciate.

Many additional bacterial metabolites, such as niacin, trimethylamine N-oxide, branched chain amino acids, indolimines, and bacterially produced succinate, to name a few, affect host physiological processes. Although these compounds have not yet been as well studied as BAs and SCFAs, they serve as a reminder that bacterial-produced metabolites are powerful signaling molecules and we are still in the infancy of understanding how the host interprets luminal secondary metabolites as environmental and dietary signals. Niacin (vitamin B3) treatment protects against HFD-induced obesity and is accompanied by decreased de novo lipogenesis, increased WAT/BAT thermogenic activity, and decreased intestinal absorption of cholesterol, triglycerides, and free fatty acids in WT, but not the niacin receptor GPR109A knockout mice.^192^ Trimethylamine N-oxide, which is purported to promote chronic diseases, such as atherosclerosis, is a downstream product of bacterial conversion of carnitine and choline-containing compounds (often found in red meats) into trimethylamine.^193^ Although still mechanistically poorly understood, the association of trimethylamine N-oxide with the risks of cardiovascular disease has been the object of rigorous investigation. Branched chain amino acids are important signaling molecules produced by mammalian hosts as well as luminal bacteria.^194^ The activating pathways play important roles, such as protein synthesis and insulin secretion, thus becoming a candidate for understanding how their modulation by bacteria affects obesity, diabetes, and cancer. Recent studies demonstrate that indolimines, which are produced by the CRC-associated bacterial species *Morganella morganii*, are capable of eliciting DNA damage.^195^ In gnotobiotic mice, *M morganii* increases intestinal permeability, transcriptional patterns associated with abnormal DNA replication, and intestinal epithelial cell proliferation.^195^ In addition, in a mouse model of CRC, indolimine-producing *M morganii* increased colonic tumor burden.^195^ Bacterially derived succinate plays an important role in intestinal inflammatory homeostasis, although is still incompletely understood.^196^ Although recognized as an intermediary of the tricarboxylic acid and potential pro-inflammatory agent, it is also substrate for GPR91/SUNCN1,^197^ which potentially has anti-inflammatory role in neural stem cells^198^ and macrophages,^199^ which could play a role in reversing the immunometabolic effects of obesity. Given that some metabolites (eg, secondary BAs and SCFAs) are only produced by bacteria, whereas others (eg, succinate and branched chain amino acids) are produced by both the host and bacteria, this makes the latter group much more difficult to study in the context of host–microbiome relationship. To further complicate matters, up to 70% of observed molecules resulting from an untargeted microbial metabolomic result from murine models remain functionally uncharacterized.^15^ Active collaboration between multiple scientific fields, including but not limited to physiologists, bioinformaticians, chemists, ecologists, and microbiologists, will be necessary to understand the contribution of microbe–host interactions and human health.

Over the past 5 years, several exceptional reviews have described the role of microbiota-derived metabolites in IBD.^13,14,200–204^ These studies have identified BAs, SCFAs, tryptophan metabolites, and sulfur-containing metabolites as being potentially implicated in the pathogenesis of IBD. The importance of FXR and TGR5 receptors in affecting the pro-inflammatory phenotype of IBD is also well-described. In addition, in humans, the newly discovered Asp-CA, CDCA; citrulline-CA, CDCA, DCA; Glu CA, CDCA; His-CA,CDCA; Ile/Leu-CA; Met-CA, Phe-CA, Thr-CA, CDCA; Trp-CA, CDCA, DCA; and Tyr-CA, CDCA are increased in IBD,^15,18,205^ thus demonstrating that our understanding of the role of BAs in the pathogenesis of IBD is still incomplete. Although compositional approaches investigating whether altering micro-organisms in patients with IBD can yield therapeutic benefits are ongoing, understanding the functional implications of these microbiome changes may yield an understanding of the pathophysiology that can lead to therapeutics that work directly on the host receptors and hence have less variability in their effects.

Fecal microbiota transplantation for ailments other than recurrent *Clostridium difficile* infections after antibiotic treatment have been disappointing and plagued with inconsistent results.^206–210^ Probiotics have not done better with inconsistent results between studies, despite a strong publication bias.^211,212^ This is likely because live bacterial products have difficulty surviving in the luminal environment. Host-mediated effects, such as peristalsis and innate and adaptive immunity, as well as competition for niche availability with native micro-organisms, prevent survival of strains exogenous to the luminal environment. This is particularly apparent with patients who have received fecal microbiota transplants when, with few exceptions, the patient’s native microflora and largely, if not completely, displace the transplanted microbiota.^213^ Understanding how to make more reliable change to the gut microbiome to affect host physiology remains a topic of active investigation. This can only occur with better understanding of microbial niches, the interrelationship of micro-organisms within the luminal environment, and the factors that promote engraftment. In addition, microbiome research focused on compositional approaches to develop potential therapeutic targets are hampered by a lack of understanding of what constitutes a “normal” microbiome. Thus, it is unclear whether most therapies that target microbiome composition have a detectable impact on the gut microbiome, or are robust to the interpersonal diversity and plasticity of the microbiome in human hosts.^7,8^ Furthermore, many different configurations of the microbiota can lead to the same functional result,^9^ suggesting that microbial functions should be the main target of therapeutic interventions. This will require a better mechanistic understanding of specific microbe–host relationships and interventions that stress the functional modulation of the gut microbiome.

Another exciting approach that has gained momentum in the past few years has been the rise of synthetic biology approaches to develop live bacterial products. Although engineered live bacterial products have been mainly tested under noncolonizing conditions (ie, gnotobiotic/germ-free mice, immunosuppressed mice, and antibiotic-treated mice), they hold tremendous potential in that they can not only lead to therapeutic drugs, but could also, in the form of engrafting beneficial bacteria, lead to agents with curative effect.^8^ Most engineered bacteria, however, have difficulty provoking functional change in hosts with an intact microbiome, including humans. This has severely limited their use as a potential therapeutic for GI diseases. To understand whether a bacterial function in the gut lumen can convey, or disrupt, a phenotype, new tools to functionally manipulate the gut microbiome will be needed. Because gut bacteria can sense and manipulate the gut luminal environment, they are attractive for engineered cell-based therapeutics. Current research paradigms for engineered cell-based therapies engineer bacteria from laboratory strains, but these strains cannot effectively colonize conventionally raised WT hosts, limiting their use in mechanistic and therapeutic studies.^8^ There is a need for the development of novel engineered bacterial therapeutics that overcome these obstacles.^214^ Developing and using engineered native bacteria to knock-in specific genes and pathways expands our ability to functionally manipulate the gut microbiome.^12^ This technique can help us assess the validity of the assumptions of functional microbiome studies, and to finally resolve many hypotheses that have been generated by the associative, compositional-based microbiome research done over the last 2 decades. Engineered native bacterial therapeutics would not require tremendous resources to modify and would allow for long-term treatment of diseases, such as the GI diseases described in this review.

## Figures and Tables

**Table 1.** Physiological Processes Affected by Bile Acid Receptor Signaling

Physiological process	Receptors	Action

Glucose homeostasis	FXR	Intestinal FXR activation induces FGF-15/FGF-19 secretion, which increases glycogenesis and inhibits GLP-1 production^30^ Intestinal FXR modulates glucose absorption and postprandial glucose utilization^215^
	TGR5	TGR5 increases GLP-1 release in intestinal L cells^30^ and increases energy expenditure in BAT and muscle^43^
BA homeostasis	FXR	Activation of hepatic FXR inhibits de novo BA synthesis in the liver^30^ FXR activation in the GI tract inhibits de novo BA synthesis in the liver via FGF- 15/FGF-19 signaling^30^ Inhibition of de novo BA synthesis increases hepatic cholesterol
Lipid homeostasis	FXR	FXR activation increases WAT browning^70^
	TGR5	TGR5 increases energy expenditure in BAT through the TGR5-cyclic adenosine monophosphate-type 2 iodothyronine deiodinase signaling pathway^43^
Insulin signaling	FXR	Pancreatic FXR positively regulates insulin synthesis and glucose-induced insulin secretion^28^
	TGR5	Pancreatic TGR5 positively regulates insulin synthesis and glucose-induced insulin secretion^216^ Intestinal TGR5 activation improves glycemic control by GLP-1 release in intestinal L cells, which increases postprandial insulin secretion from pancreatic *β* cells^216^
Inflammation	FXR	FXR activation inhibits inflammatory cytokine production in the GI tract and improves intestinal barrier integrity^217^
	TGR5	TGR5 activation protects against lipopolysaccharide-induced inflammation^218^

## Abbreviations used in this paper:

BA: bile acid
BAT: brown adipose tissue
BSH: bile salt hydrolase
CA: cholic acid
CDCA: chenodeoxycholic acid
CRC: colorectal cancer
DCA: deoxycholic acid
FGF: fibroblast growth factor
FXR: farnesoid X receptor
GI: gastrointestinal
HDAC: histone deacetylase
HFD: high-fat diet
IBD: inflammatory bowel disease
IBS: irritable bowel syndrome
IBS-C: constipation-predominant irritable bowel syndrome
IBS-D: diarrhea-predominant inflammatory bowel disease
MS: mass spectrometry
NAFLD: nonalcoholic fatty liver disease
NASH: nonalcoholic steatohepatitis
PD: Parkinson’s disease
SCFA: short-chain fatty acid
T*β*MCA: tauro-*β*-muricholic acid
TUDCA: tauroursodeoxycholic acid
UDCA: ursodeoxycholic acid
WAT: white adipose tissue
WT: wild-type
